# Bathymetric Variation in Recruitment and Relative Importance of Pre- and Post-Settlement Processes in Coral Assemblages at Lyudao (Green Island), Taiwan

**DOI:** 10.1371/journal.pone.0081474

**Published:** 2013-11-27

**Authors:** Yoko Nozawa, Che-Hung Lin, Ai-Chi Chung

**Affiliations:** Biodiversity Research Center, Academia Sinica, Taipei, Taiwan, ROC; College of Charleston, United States of America

## Abstract

Studies on coral communities have typically been conducted in shallow waters (∼5 m). However, in the face of climate change, and as shallow coral communities become degraded, a greater understanding of deeper coral communities is needed as they become the main reef remnants, playing a central role in the future of coral reefs. To understand the dynamics of deeper coral assemblages, the recruitment and taxonomic composition of different life-stages at 5 and 15 m depths were compared at three locations in Lyudao, southeastern Taiwan in 2010. Coral recruits (<1 cm diameter, <4 months old) were examined using settlement plates. Juvenile corals (1–5 cm, several years old) were examined with quadrats, and adult corals (>5 cm, several years to decades old) were examined using transect lines. Pocilloporid and poritid corals had similar and higher numbers of recruits at 5 m compared to 15 m, whereas acroporid recruits were more abundant at 15 m. The primary cause for the former may be larval behavior, such that they position themselves in shallow waters, while that for the latter may be the dominance of brooding acroporid species (*Isopora* spp.) at 15 m. The taxonomic composition, especially between recruits and juveniles/adults, was more similar at 15 m than at 5 m. These results suggest a change in the relative importance of pre- and post-settlement processes in assemblage determinants with depth; coral assemblages in shallow habitats (more disturbed) are more influenced by post-settlement processes (mortality events), while those in deeper habitats (more protected) are more influenced by pre-settlement processes (larval supply).

## Introduction

Many coral communities have deteriorated as a result of various anthropogenic disturbances, including climate change, and further change is predicted in the future [1], [2]. This ongoing trend of deterioration, mainly observed in shallow coral communities, has directed the attention of researchers to deeper coral communities, which are thought to inhabit less disturbed conditions [3]–[6] and are therefore likely to survive better in an increasingly hostile environment in the future [7]–[10]. If this prediction is true, deeper coral communities would become the main remnants of future coral reefs, playing a more central role than degraded shallow coral communities, and may also serve as a source of coral recruits for the recovery of shallow coral communities [7], [9], [10]. Despite a growing need for information concerning deeper coral communities, this knowledge is limited since coral studies have mainly been conducted in shallow waters (∼5 m) [10]. Therefore, information on deeper coral communities is valuable, especially information related to their dynamics and interactions between shallow and deep corals.

With recent advances in deep-water survey technologies, new studies of deeper coral communities have begun. However, most of these studies are focusing on “mesophotic coral communities” that occur deeper than 30 m [8], [10], and studies of coral communities on reef slopes at 10–30 m depth have not been given equal attention. Given the dramatic change in environmental gradients in the upper 10–20 m of water, especially regarding light intensity and wave action, and associated biotic/abiotic changes with depth (e.g., movement of sand gravels, algal abundance, herbivores and coral growth) [3]–[5], [11], [12], the dynamics of deeper coral communities (10–30 m) cannot be assumed to be the same as those of shallow coral communities (∼5 m). In fact, coral assemblages at 10–30 m depth possess the highest coral diversity, presumably due to the less disturbed conditions, lower competition for space and sufficient larval supply [4].

The objective of this study was to investigate the recruitment process of deeper coral assemblages to understand their dynamics at Lyudao, southeastern Taiwan. Coral recruitment was examined using settlement plates at 5 m and 15 m depth. Data on the taxonomic composition of juvenile (1–5 cm) and adult corals (>5 cm) were also collected to examine changes in assemblage structure among different life stages (recruits, juveniles and adults). In Southeast Asia, coral recruitment patterns in deeper water (>10 m) have been measured in only a single location (Okinawa, Japan) [13]–[15], and most studies at similar depths have been undertaken in other regions of the world [6], [12], [16]–[23]. The aforementioned studies generally found a higher density of coral recruits at 10–20 m, and argued the importance of the pre-settlement process (larval supply) in relation to the richer coral assemblages at this depth range [4]. In the present study, we observed that coral recruitment varied with depth and that this variation also differed among coral taxa. We also found that similarities in taxonomic composition among the three life-stages varied with depth, and argue that the relative importance of pre- and post-settlement processes in assemblage determinants may vary with depth.

## Materials and Methods

### Study site

**Figure 1:**
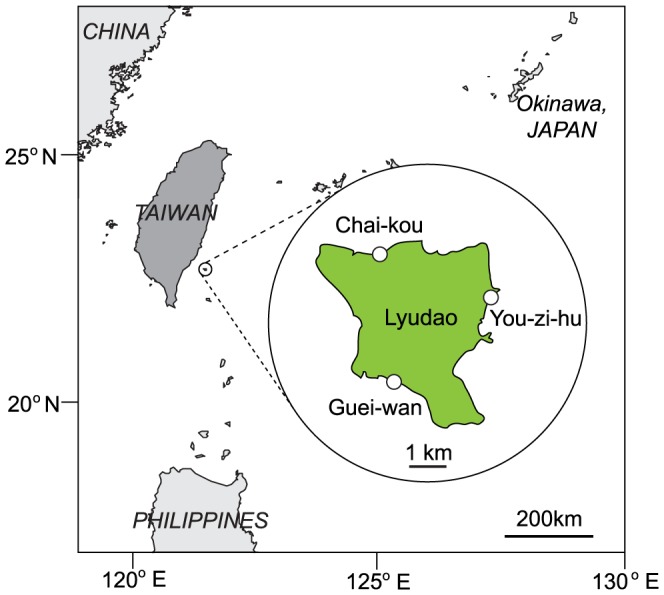
Study location. Coral assemblages at 5(Chai-kou, Guei-wan, You-zi-hu) around Lyudao (Green Island), Taiwan in 2010.

This study was conducted in 2010 at 5 m and 15 m depth on reef slopes at three locations (Chai-kou, Guei-wan, You-zi-hu) around Lyudao (Green Island), southeastern Taiwan (Fig. 1). Permits for this study were granted by Taitung county government. At each depth/site combination, surveys were performed in an area of ∼50×50 m. Lyudao is an offshore islet located in the middle of the Kuroshio Current [24] that may transport various marine organisms, including coral larvae, from the up-current coral triangle area. Lyudao is surrounded by clear water, and has well-developed fringing reefs to ∼30 m depth containing approximately 250 scleractinian coral species [25]. Several tropical typhoons pass through the vicinity of Lyudao every year, causing significant disturbance of its marine biota [26], [27].

### Coral recruitment survey

Settlement plates with a “refuge structure” were used to assess coral recruitment. Although settlement plates have been used in coral recruitment studies since the 1970s, most settlement plates have consisted of flat surfaces [except 23], without refuge structures like crevices, pits, and grooves. These substrata often result in no or low recruitment on exposed, upward plate surfaces most likely due to grazing by herbivores [22], [28], [29]. Previous studies that examined settlement plates with refuges found more coral recruits with a higher taxonomic diversity compared to the traditional settlement plates with plain surfaces [[30], [31], Nozawa, Y. unpublished data]. As coral species with small-sized and/or slow-growing recruits are predicted to depend more heavily on refuge structure for post-settlement survival [32], the use of settlement plates with refuges is expected to provide less biased and more artifact-free results in comparison with a plain substratum which is uncommon in coral reef habitats. For this study, we used commercially available unglazed terracotta plates (10 cm×10 cm×2 cm) with two grooved surfaces (14 grooves surface^−1^, groove size: 5 mm wide, 100 mm long, 2 mm deep). The dimensions of the refuge structure on the settlement plates were determined according to Nozawa [33]. By using settlement plates with refuges in this study, we had more numerous and taxonomically diverse coral recruits than in a previous study using plain settlement plates conducted at the same location [34].

At each depth/site combination, 15–18 settlement plates were deployed haphazardly. Settlement plates were fixed to the sea bottom a few centimeters above the substrata using stainless bolts and nuts. To avoid sediment deposition filling the refuges on plate surfaces and negating their effect [28], settlement plates were fixed at an angle of ∼45° to the bottom.

Settlement plates were deployed in early April, approximately 2 to 3 weeks before the main coral spawning period (April–June) [[35], Y. Nozawa unpublished data] in order to biologically condition the plate surfaces. Settlement plates were retrieved 4 months later to cover the main period of coral recruitment predicted for southern Taiwan. The number of settlement plates retrieved at the 5 m and 15 m sites was as follows, respectively: for Guei-wan, 17 and 18; for Chai-kou, 15 and 15; for You-zi-hu, 16 and 16. Environmental data (temperature and light intensity depth^−1^ site^−1^) were collected during the period in which settlement plates were deployed in 2010, using HOBO pendant temperature/light data loggers (Onset Computer Corp., USA). Retrieved settlement plates were soaked in a dilute chlorine bleach to remove algae and soft-bodied epibenthos. Coral recruits (skeletons) on the top and bottom plate surfaces were counted under a stereomicroscope and taxonomically identified into four family groups (Acroporidae, Pocilloporidae, Poritidae, and others) according to Babcock et al. [36].

### Coral juvenile and adult assemblage survey

Coral juvenile and adult assemblage surveys were performed in the same areas used for the deployment of settlement plates at 5 m and 15 m depths at the three sites. Quadrats (25 cm×25 cm) were used to assess juvenile corals. At each depth/site combination, 28–71 quadrats were haphazardly placed on rocky substrata. Juvenile corals (1–5 cm in diameter) that appeared in the quadrats were photographed with a scale and taxonomically identified later. Adult coral (>5 cm in diameter) surveys were performed using a line intercept method with 10-m lines. At each depth, six transects were placed haphazardly along the depth contour. All scleractinian corals below the lines were photographed along with the line and a scale and taxonomically identified later. For each individual, the length of the individual that was intercepted by the line was measured to obtain a cover estimate, and the maximum width of the individual perpendicular to the line was measured for the density estimate [37]. Density was calculated using the following equation:
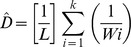
where 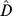 =  estimate of population density, 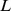 =  length of all lines combined, 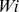 =  perpendicular width of individuals intersected, 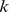 =  the total number of individuals intercepted on all lines. We separated taxa of juveniles and adult corals into the four family groups (Acroporidae, Pocilloporidae, Poritidae and others), in which the genus *Alveopora* was allocated to Acroporidae following a recent taxonomic revision [38].

### Environmental conditions

**Figure 2:**
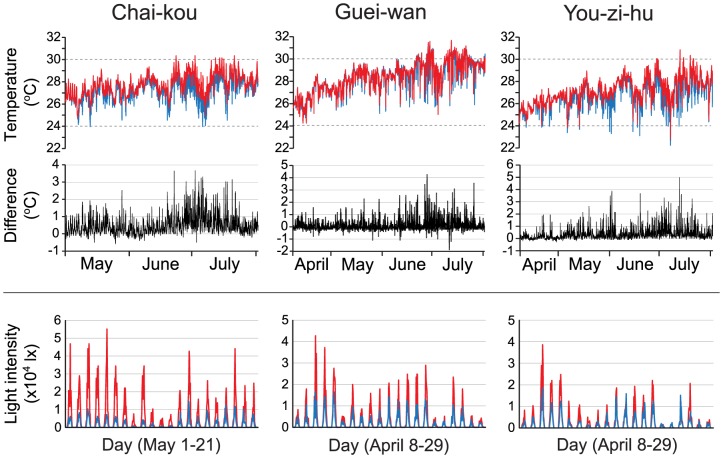
Environmental condition. Seawater temperature and light intensity were monitored in 2010 at the 5(red) and the 15 m (blue) sites at three study locations (Chai-kou, Guei-wan, You-zi-hu). For temperature, data were obtained during the 4-month deployment of the settlement plates for the coral recruitment surveys. Absolute differences in temperatures between the 5 m and the 15 m sites are shown below the temperatures. For light intensity, data from the first 3 weeks of the deployment period are shown as the values then gradually declined due to the gradual coverage of the sensor component of the loggers by benthic organisms. Data for Chai-kou started from May due to the loss of initial data-loggers in April.

Seawater temperature and light intensity were measured at 5 m and 15 m depths during the major recruitment season (April–July) in 2010 (Fig. 2). The temperature was higher at 5 m, and the maximum temperature difference between the two depths reached up to 5°C. However, in most cases (>85% of data), the temperature difference was <1°C. The median (25^th^ and 75^th^ percentiles) of the temperature difference was 0.4°C (0.1∼0.7) in Chai-kou, 0°C (−0.1∼0.2) in Guei-wan and 0.2°C (0.1∼0.5) in You-zi-hu. Light intensities at 15 m (max; 4–5×10^4^ lx) were about four to five times lower than those at 5 m (<1×10^4^ lx).

### Statistical analysis

The number of coral recruits per settlement plate (recruits on top and bottom surfaces were pooled) was analyzed using a generalized linear model (GLM) with a Poisson error distribution by the glm function in R (version 3.0.0) [39]. In the GLM, sites and depths were treated as a fixed factor. Pairwise post-hoc comparisons were performed with a Tukey test using the glht function in the package multcomp (version 1.2–17) in R. The same statistical analyses were applied for acroporid, pocilloporid and poritid recruits, respectively.

Similarities in taxonomic composition between coral recruits, juveniles and adult assemblages at 5 m and 15 m were visualized using non-metric multidimensional scaling (MDS) based on Bray–Curtis similarity coefficients with relative density data. A one-way analysis of similarity (ANOSIM) was conducted to determine the significance of any observed differences in taxonomic composition between the three life-stages at each depth [40]. The MDS analysis and ANOSIM test were performed using Primer software, version 6 (Primer-E Ltd, Plymouth, UK).

## Results

### Coral recruitment

**Figure 3:**
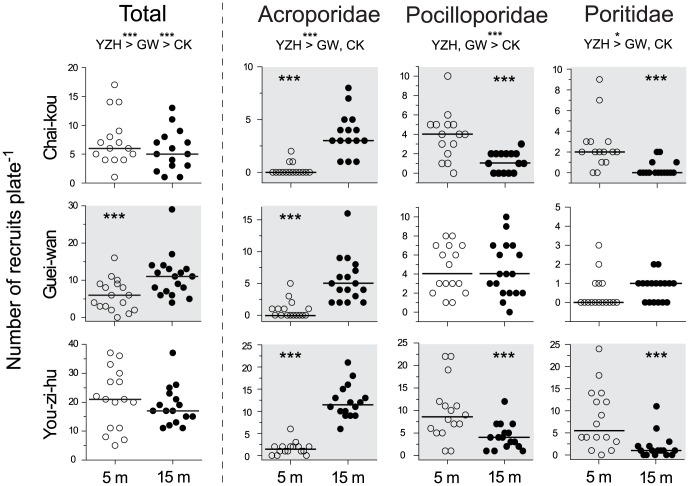
Coral recruits at two depths. The numbers of coral recruits per settlement plate at the 5(open circle) and the 15 m sites (closed circle) are shown. The horizontal bar denotes the median. The data were analyzed using a generalized linear model (see the method section for details), and results with a significant difference are highlighted with a grey background (p<0.001). Results on the comparison of recruit numbers between the sites are also presented for each taxonomic group below the heading; CK = Chai-kou, GW = Guei-wan, YZH = You-zi-hu; *** p<0.001, * p<0.05.

Results on the number of coral recruits per settlement plate are summarized in Figure 3. Of the 97 settlement plates retrieved, most settlement plates (>80%) had more coral recruits on the upward plate surfaces. Among the three sites, You-zi-hu generally had the highest number of recruits, followed by Guei-wan and Chai-kou, in the four recruit categories examined (Fig. 3). Comparison between the two depths (5 m and 15 m) revealed no significant difference in the total number of recruits at Chai-kou and You-zi-hu; however, at Guei-wan more recruits were observed at 15 m (GLM: p<0.001). In the family level analyses, a higher number of acroporid recruits was recorded at 15 m at all three sites (GLM: p<0.001), whereas a higher number of pocilloporid and poritid recruits was recorded at 5 m in Chai-kou and You-zi-hu (GLM: p<0.001); the number of these recruits at Guei-wan was similar at both depths.

### Taxonomic composition of recruits and later life stages

**Figure 4:**
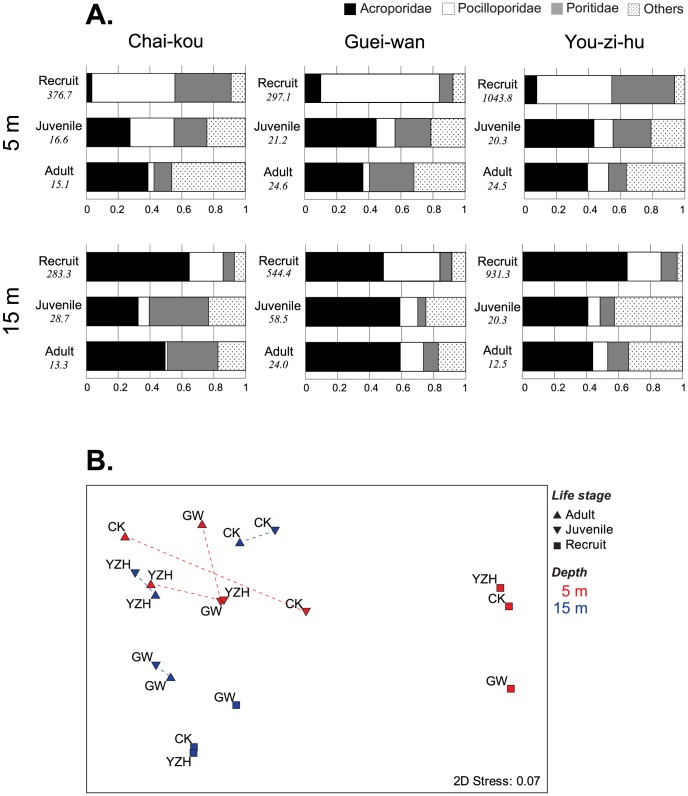
Taxonomic composition of three life-stages at two depths. **A**) Relative densities of the three dominant families at three life-stages (recruits, juveniles and adults) are shown for the 5 m (above) and 15 m sites (below) at three locations. The number on the left-hand side of each bar denotes the density (m^−2^). **B**) Multi-dimensional scaling (MDS) ordination, based on Bray–Curtis similarity coefficients, for the relative density data in Fig. 4A. Juvenile and adult stages of each site are connected by a dashed line. Data for the 5 m sites are shown in red, and data for the 15 m sites are in blue. CK = Chai-kou, GW = Guei-wan, YZH = You-zi-hu.

Relative abundance data (in density) showed that the dominant recruit groups at 5 m were pocilloporids and poritids (ca. 80% of recruits), whereas those at 15 m were acroporids (48–65%), followed by pocilloporids (12–38%) (Fig. 4A). In comparison with those of juvenile and adult corals, a distinct difference was observed between the recruit and juvenile stages at 5 m, followed by more moderate changes between the juvenile and adult stages. The overall trend at 5 m was that, from the recruit to adult stages, the proportions of pocilloporids and poritids decreased, while those of acroporids and others increased. In contrast, at 15 m, the composition was more similar between the three life-stages, especially at Guei-wan. This observation was supported by the MDS plot of the relative density data, which showed two distinct groups, i.e., coral recruits at 5 m and others (Fig. 4B). Within the group of others, recruits at 15 m were also grouped together, and juvenile and adult assemblages at 15 m were located closer together (i.e., were more similar) than those at 5 m. The ANOSIM results showed a significant difference among the three life-stages at both 5 m and 15 m with a larger R statistic value (i.e., larger difference) at 5 m (5 m: R = 0.745, p<0.01; 15 m: R = 0.407, p<0.05).

## Discussion

### Recruitment patterns and water depth

Densities of coral recruits were similar at the 5 m and 15 m sites. However, when data were partitioned by family, family-level analyses revealed variations in recruitment patterns between depths. Most acroporid recruits occurred at 15 m depth, while a similar number or more pocilloporid and poritid recruits occurred at 5 m depth.

*Isopora* species that release planula larvae (i.e., brooders) dominated adult acroporid assemblages at 15 m depth (16–58% of acroporids), and their densities (1–7.6 m^−2^) were 5–19 times higher than those at 5 m depth (0.2–0.7 m^−2^). This result would largely account for the higher number of acroporid recruits at the 15 m sites, assuming that many were *Isopora* recruits, because a strong correlation between density of adults and recruits is common in brooding corals [30], [41], [42].

In contrast, the higher recruitment densities of pocilloporids and poritids at 5 m could not be explained by adult distribution as densities were similar at both depths [0.6–3.2 m^−2^ (5 m) and 0.2–3.5 m^−2^ (15 m) for Pocilloporidae, 1.7–6.8 m^−2^ (5 m) and 1.6–4.2 m^−2^ (15 m) for Poritidae], and the dominant species of the two families were spawners in Lyudao (*Pocillopora verrucosa*, *P. eydouxi* and massive *Porites* spp.; Y. Nozawa, unpublished data). A potential cause of the recruitment patterns with depth may be larval behavior. Previous studies have reported positive phototaxis and/or negative geotaxis in planula larvae of several coral species, including two pocilloporid species, *P. damicornis* and *Seriatopora hystrix* [43]–[46]. With a rapid reduction in light intensity with depth, the larval swimming behavior could have created a negative depth gradient in larval supply, enhancing recruitment at the shallow sites [44]. Planula larvae of some coral species are also known to show depth-dependent settlement behavior in response to benthic communities [15], [47] and a certain light environment [48]–[50]. Similarly, it is possible that the larval behavior of acroporids, in addition to the depth gradient of adult *Isopora* density, influenced their recruitment pattern (either enhancing or weakening it).

As an alternative explanation, variation in recruit mortality with depth could have created the same recruitment patterns for the three dominant families. However, this explanation is less likely because previous studies rejected the hypothesis of different mortality between depths (0–11 m) [14], [48], [51]. A recent genetic study also supported the larval behavior hypothesis, demonstrating some evidence of larval migration from deep to shallow habitats in *S. hystrix* [7].

The pattern of higher recruitment of pocilloporids and poritids at shallow sites (<6 m) has also been reported by several previous studies [21], [23] but many found higher recruitment at 10–20 m [16]–[19], [52]. These studies attributed the decline in recruits at shallow depths to higher post-settlement mortality caused by intense grazing of herbivores [cf. 22]. Of these studies, Wallace [23] and the present study are the only studies using settlement substrata with refuges that protect coral recruits from grazers, while others used plain settlement plates. It is therefore likely that the decline in recruits at shallow sites was caused by an absence of refuge structure on the settlement substrata used in previous studies [28,30,31, Y. Nozawa, unpublished data]. Given the complex surface structure seen on natural substrata in coral reef habitats, the recruitment pattern detected by settlement substrata with refuges may better reflect natural patterns.

### Assemblage determinants and water depth

Previous studies have demonstrated the importance of pre- and post-settlement processes in determining coral assemblage structure [4], [5], [13]–[16], [44], [47], [51], [53]–[56]. In the present study, we found that the relative importance of pre- and post-settlement processes changed with depth in coral assemblages. At the shallow sites, the large change in assemblage structures among the three life-stages, especially when comparing recruits and juveniles, suggested that post-settlement processes (mortality events) had a strong influence, whereas at the deep sites, the less prominent difference among the life-stages suggested a prevalence of pre-settlement processes (larval supply and behavior).

Higher disturbance frequencies and competition in shallower habitats are common on most reefs [5], [12], [16], [44], [55], and are attributed to the fact that the richest coral species-diversity occurs at 10–30 m [4]. In Taiwan, typhoons are generally the most serious natural disturbance affecting shallow reefs, and three to four typhoons typically impact the study locations each year [26], [27]. Strong wave action created by typhoons causes serious damage to shallower coral assemblages [27], [53], [57]. In particular, the movement of sand gravels that wear down coral tissues during typhoons and smother coral recruits and juveniles after typhoons has been implicated in the high coral mortality of early life-stages in shallow reef habitats [5], [53], [58]. In Lyudao, the 5 m sites were dominated by encrusting acroporid corals (mainly *Montipora* spp.) and domed faviid corals, while various other corals inhabited the 15 m sites, including tabular and branching corals. As the disappearance of non-encrusting corals from shallow habitats is commonly observed after typhoons [[59], Nozawa, Y. unpublished data], the success of the dominant coral taxa at the 5 m sites in Lyudao may largely be attributable to the frequent typhoon disturbance typical in Taiwanese waters.

Connell et al. [53] concluded that “the dynamics of coral assemblages can be understood through the variation in types and scales of disturbances and other ecological processes where disturbances are rare”. This may be applicable to the dynamics of coral assemblages at different depths, which are exposed to a negative depth gradient of disturbance, and may explain the change with depth in assemblage determinants. Although this observed change in assemblage determinants with depth may be a generality at most reef sites, as the negative depth gradient of disturbance is a general pattern [4], the conclusion of Connell et al. [53] also suggests that the relative importance of pre- and post-settlement processes at each depth may vary (i.e., are not fixed), depending on the variation in type and scale of disturbance. When (and where) disturbances reach deeper habitats, the influence of post-settlement mortality may prevail over depth, and vice versa.
